# Association between healthy beverage index and healthy beverage score with metabolic syndrome: a cross-sectional study

**DOI:** 10.1017/jns.2024.65

**Published:** 2025-02-24

**Authors:** Kimia Leilami, Zahra Mahmoudi, Zahra Ghazimpradi, Mehran Nouri, Atefeh Torabi Ardekani, Fariba Moradi Ardekani, Morteza Zare, Seyed Jalil Masoumi

**Affiliations:** 1 Nutrition Research Center, School of Nutrition and Food Science, Shiraz University of Medical Sciences, Shiraz, Iran; 2 Department of Nutrition, Science and Research Branch, Islamic Azad University, Tehran, Iran; 3 Department of Nutrition, Qazvin university of medical science, Qazvin, Iran; 4 Health Research Institute, Babol University of Medical Sciences, Babol, Iran; 5 Center for Cohort Study of SUMS Employees’ Health, Shiraz University of Medical Sciences, Shiraz, Iran

**Keywords:** Healthy beverage index, Healthy beverage score, Metabolic syndrome, MetS, Metabolic Syndrome, SUMS, Shiraz University of Medical Sciences, HBI, Healthy Beverage Index, HBS, Healthy Beverage Score, SSB, Sugar-Sweetened Beverage, CVD, Cardiovascular Disease, HDL, High-Density Lipoprotein, T2D, Type 2 Diabetes, WC, Waist Circumference, HC, Hip Circumference, BMI, Body Mass Index, FFQ, Food Frequency Questionnaire, PCA, Principal Component Analysis, WI, Well-Being Index, SES, Socioeconomic Status, IPAQ, International Physical Activity Questionnaire, ATP III, Adult Treatment Panel III, NCEP, National Cholesterol Education Program, ANP, Atrial natriuretic peptide

## Abstract

Metabolic syndrome (MetS) is a widespread and complex health disorder. Dietary habits and consumption of simple sugars have been shown to play an important role in the prevention and treatment of MetS. This cross-sectional study was conducted in a population of 3380 adults from the Shiraz University of Medical Sciences (SUMS) employees’ health cohort. The healthy beverage index (HBI) and healthy beverage score (HBS) were calculated. Risk for MetS and its components, including blood pressure, fasting blood glucose, waist circumference, triglyceride levels, and high-density lipoprotein cholesterol, were measured using standardised protocols. Results showed a significant inverse association between higher adherence to HBI (OR = 0.60, 95% CI: 0.48–0.74, *P* < 0.001) and HBS (OR = 0.80, 95% CI: 0.65–0.97, *P* = 0.030) with lower risk of MetS. Also, we observed a significant association between higher level of HBI and HBS with decreased risk of hypertension, as a critical component of MetS. These findings support the notion that healthier beverage consumption, as indicated by higher HBI and HBS levels, may play a critical role in reducing the risk of MetS.

## Introduction

The metabolic syndrome (MetS) is a collection of metabolic abnormalities, including central obesity, dyslipidemia, hypertension, and insulin resistance.^(1)^ Approximately 40–46 per cent of world’s adult population have the risk factor for MetS.^(2)^ The prevalence of MetS in the Asia-Pacific region varies by region, age group, and gender, with 25% of the population affected.^(3)^ MetS is strongly associated with increased risk of cardiovascular disease (CVD) and type 2 diabetes (T2D).^(4)^ Consequently, the increasing prevalence of MetS worldwide has become a significant public health concern.^(5,6)^

Lifestyle changes, such as quitting smoking, increasing physical activity, and especially reducing consumption of processed meat and sugar-sweetened beverages (SSBs), have been shown to be effective in preventing and treating MetS.^(7,8)^ In this study, our primary objective is to investigate the relationship between beverage quality and the prevalence of MetS, aiming to uncover potential dietary factors that could influence metabolic health. Beverage consumption is a critical aspect of dietary habits and is associated with MetS. The type and amount of beverages consumed can significantly affect overall nutrient intake and health outcomes.^(9)^ Previous studies have shown that SSBs are positively associated with MetS and its components.^(10,11)^ Conversely, some studies have shown that higher consumption of non-caloric beverages such as tea and coffee is associated with a lower risk of MetS and its components.^(12,13)^ However, another study found no significant association between coffee consumption and MetS.^(14)^

Nevertheless, there is no consensus on how to define and measure healthy beverage consumption. Some studies have used a simple classification of SSBs versus non-SSBs, while others have taken a more comprehensive approach, such as the healthy beverage score (HBS) or the healthy beverage index (HBI).^(15,16)^ HBS and HBI are scoring systems that assign points to different beverage types based on their health benefits, including nutrient content and potential health benefits. Higher scores indicate higher consumption of healthier beverages such as water, tea, and low-fat milk and lower consumption of less healthy beverages such as SSBs. Therefore, they can be appropriate tools for evaluating the quality of beverage consumption and its association with various diseases, as they comprehensively consider both the quality and quantity of all beverage types and utilise a complex scoring system.^(16,17)^

To date, few studies have examined the association between HBI and MetS, while most studies focusing on the effects of only one type of beverage, and no research has yet examined the association between HBS and MetS. Recently, M.Nouri et al. demonstrated that maybe a higher HBI score is related to a lower risk of MetS.^(18)^ Also, another cross-sectional study found that higher HBI can be related with lower risk factors of MetS among over weigh and obese individuals.^(19)^ However, the big difference with the current study is that we additionally evaluated HBS to receive more comprehensive results and we encompassed a more diverse population.

## Methods

### Participants and study design

This study was conducted according to the guidelines laid down in the Declaration of Helsinki, and all procedures involving human subjects were approved by the local ethics committee of Shiraz College of Medical Sciences (code: IR.SUMS.SCHEANUT.REC1402.002). Written informed consent was obtained from all subjects, and they are accessible.

The study population included adults who participated in the Shiraz University of Medical Sciences Employees’ Health Cohort Study (SUMS EHCS), conducted between 2018 and 2019, is a comprehensive research endeavour designed to provide insights into various aspects of employee health. This longitudinal study encompasses a diverse range of participants from different occupational backgrounds within the university setting. With meticulous attention to detail and adherence to rigorous research protocols.

Under the guidance of Dr. Masoumi, a renowned expert in the field of nutrition, the SUMS EHCS places particular emphasis on dietary factors and beverage intake in relation to overall health outcomes. Dr. Masoumi’s expertise ensures that the study design incorporates robust methodologies for the collection and analysis of dietary data, thereby enhancing the validity and reliability of the findings. The cohort study included participants aged 20 to 65 who were officially or contractually employed at Shiraz College of Medical Sciences. After excluding 1,170 individuals who either had missing data on any MetS components, struggled to understand the questions, did not participate in the survey, were menstruating or pregnant, or reported a daily energy intake outside the range of 800 to 4,200 kcal, A total of 3,380 participants were included in the statistical analysis.

### Anthropometric assessment

Anthropometric measures, including weight, height, body mass index (BMI), waist circumference (WC), and hip circumference (HC), are essential for evaluating obesity and its association with MetS. Weight was measured with minimal clothing and no shoes on a scale with an accuracy of 0.1 kg. Height was measured using a standard method with an accuracy of 0.1 cm while participants stood with shoulders, hips, and legs against the wall, without shoes. BMI was calculated by dividing weight by height squared (kg/m^2^). WC was measured at the narrowest part of the WC and HC was measured at the widest part of the hip using a rigid tape with an accuracy of 1.0 cm.^(20)^ In addition, body composition data were obtained using the BIA InBody 770.^(21)^

### Dietary intake, HBI and HBS

Food intake information was collected using a modified semi- 125 item food frequency questionnaire (FFQ) based on Iranian foods. The validity and reliability of the questionnaire had been confirmed in previous internal and external studies.^(22,23)^ Trained interviewer (A.Torabi Ardekani) administered the questionnaire, and participants reported their consumption of each food item daily, weekly, monthly, or annually during the past year. Energy and nutrient intakes were calculated using the adapted version of NUTRITIONIST IV for Iranians (version 7.0; N-Squared Computing, Salem, OR, United States).

Beverage intake was determined using the FFQ about food beverage like tea, coffee, high-fat and low-fat milk, soda, fruit juices, and additional questions on water intake in different seasons. HBS and HBI were calculated based on beverage consumption according to methods previously published by Dufey and Davy.^(15)^ HBI divided beverages consumed into eight groups, including water, tea, coffee without sugar, low-fat milk, high-fat milk, natural fruit juices, SSBs, diet beverages, and alcoholic beverages. The index was reported on a scale of 0 to 100, with a higher value indicating healthier beverage consumption. In the Iranian population, in which alcoholic and diet beverages were not consumed, the final HBI value ranged from 0 to 90.^(24)^ The HBS included seven components that were classified as adequate intake (low-fat milk, tea, and coffee without sugar) and moderate intake (high-fat milk, SSBs, diet beverages, fruit juice, and alcoholic beverages). Each component was assigned a score from 1 to 4 depending on consumption, resulting in a total score from 7 to 28. Because data on the consumption of alcoholic beverages and diet drinks were not available in this study, the HBS ranged from 5 to 20.^(25)^

### Covariates

Covariates included sex, age, education level, marital status, number of children, smoking history, hookah use, drug and alcohol use, and clinical illness. Data on chronic diseases and medication use were also collected. Socioeconomic status (SES) was assessed using a well-being index (WI) based on household items and amenities through principal component analysis (PCA). The WI was divided into tertiles, with the first tertile representing the lowest income category and the last tertile representing the highest income category.

### Physical activity

Physical activity data were collected using the International Physical Activity Questionnaire (IPAQ), which assesses the frequency and intensity of various activities. Participants were categorised into three activity levels: light, moderate, and intense.^(26)^

### MetS and biochemical assessment

MetS was defined according to National Cholesterol Education Program (NCEP) Adult Treatment Panel criteria III (ATP III) and requires the presence of at least three of the following five components: central obesity, elevated triglycerides, decreased high-density lipoprotein (HDL) cholesterol, elevated blood pressure, and elevated fasting blood glucose. Blood pressure was measured with a blood pressure monitor after at least 15 minutes of rest, and fasting blood samples were collected for biochemical analyses, including fasting blood glucose and lipid profile.^(27)^

### Statistical analysis

Statistical analysis was performed with SPSS software (version 26.0, Inc, Chicago, IL), and a significance level of *P* < 0.05 was considered statistically significant. Because of the nonnormal distribution of variables in the population, nonparametric tests were performed. Baseline characteristics were compared with Mann-Whitney and chi-square tests for continuous and categorical variables, respectively. SES was indicated by the PCA-derived wealth index.^(28)^ The Mann-Whitney test was used to compare intake of macronutrients and HBI components between groups with and without MetS. Univariate logistic regression was used to assess the relationship between the tertiles of HBI and HBS with the probability of MetS. Multivariate analysis was performed using the backward LR method to account for the effects of confounding variables (variables with *P* < 0.25 in the univariate analysis assume cofounder in multivariate analysis). Univariate and multivariate analyses were also performed to assess the relationship between HBI and HBS tertiles with each component of MetS.

## Results

This cross-sectional study included a total of 3,380 participants, of whom 876 had MetS and 2,504 did not. Table 1 shows the demographic and baseline information of the two groups separately. The median age in the MetS group was 45 years, whereas it was 44 years in the without MetS group. The median BMI was 25.71 in the MetS group and 28.39 in the group without MetS.

**Table 1.** The basic characteristic of the study population

	Metabolic syndrome		
Variable	Yes (*n* = 876)	No (*n* = 2504)	*P*-value
Age (year)^1^	45.00 (11.00)	44.00 (10.00)	**<0.001**
Physical activity (met/per week)^1^	2772.00 (6048.38)	2877.75 (5659.50)	0.607
BMI (kg/m^2^)^1^	25.71 (4.54)	28.39 (5.12)	**<0.001**
WC (cm)^1^	91.50 (11.50)	100.00 (12.80)	**<0.001**
HC (cm)^1^	105.30 (9.28)	101.00 (8.80)	**<0.001**
Average SBP (mmHg)^1^	104.66 (14.67)	92.66 (13.33)	**<0.001**
Average DBP (mmHg)^1^	88.00 (12.00)	77.50 (12.50)	**<0.001**
Blood glucose (mg/dl)^1^	96.44 (19.65)	88.41 (11.39)	**<0.001**
TG (mg/dl)^1^	165.83 (109.54)	108.64 (68.91)	**<0.001**
Cholesterol (mg/dl)^1^	187.97 (49.38)	169.30 (41.38)	**<0.001**
HDL (mg/dl)^1^	50.37 (13.65)	47.38 (13.50)	**<0.001**
Body fat percent^1^	35.45 (11.50)	33.90 (12.58)	**<0.001**
Body fat index^1^	10.00 (4.40)	8.50 (4.10)	**<0.001**
Body fat free index^1^	18.40 (3.40)	16.80 (3.10)	**<0.001**
HBS total^1^	14.00 (4.00)	14.00 (4.00)	**0.008**
HBI total^1^	67.00 (4.00)	67.00 (8.00)	**<0.001**
Gender, Male %^2^	57.1	40.5	**<0.001**
Marital status, married %^2^	89.8	84.3	**>0.001**
SES %^2^			**0.041**
Low	36.1	31.6	
Moderate	33.4	34.6	
High	30.5	33.8	
Cigarette smoking, Yes %^2^	13	8.2	**<0.001**
Hookah use, Yes %^2^	14.8	10.3	**0.001**
Yes %^2^ Opium use,	1.6	1.3	0.508
Alcohol use, Yes %^2^	5.8	3.7	**0.011**
Education, higher than diploma %^2^	90.0	92.5	**0.018**
Medication and supplementation, Yes %	52.2	58.8	**0.001**

Abbreviation: BMI, body mass index; WC, waist circumference; HC, hip circumference; SBP, systolic blood pressure; DBP, diastolic blood pressure; HDL-c, high density lipoprotein cholesterol; MUFA, mono unsaturated fatty acid; PUFA, poly unsaturated fatty acid; HBS, healthy beverage score; HBI, healthy beverage index; SES, socioeconomic status.

^1^Using Mann–Whitney for abnormal continuous variables.

^2^ Using chi-square test for categorical variables.

Values are median (IQR) for continuous and percentage for categorical variables.

Table 2 shows the mean energy intake in the MetS population, which was approximately 2146 kcal compared to 2035 kcal in the without MetS group. When the HBI components were examined, the median SSB intake was 33.86 cc in the MetS group and 24.10 cc in the without MetS group, and the difference was statistically significant. In addition, the total energy intake of beverages was 115.39 kcal in the MetS group and 106.88 kcal in the group without MetS. While, the median intake of high-fat milk was the same in both groups (65.53 ml), but the interquartile range was significantly higher in the MetS group, indicating higher consumption of high-fat milk in individuals with MetS. Also, participants with MetS had higher coffee and tea consumption than the group without MetS.

**Table 2.** Dietary nutrient intake and Healthy beverage index components between population with and without Metabolic Syndrome

	Metabolic syndrome		
Variable	Yes (*n* = 876)	No (*n* = 2504)	*P*-value
**Macronutrients intake**			
Energy (Kcal/d)	2146.40 (932.00)	2035.92 (766.00)	**<0.001**
Protein (g/d)	73.82 (35.09)	68.88 (27.60)	**<0.001**
Carbohydrate (g/d)	340.00 (159.22)	319.24 (129.72)	**<0.001**
Fat (g/d)	57.86 (29.83)	56.66 (25.49)	0.200
Total fibre (g/d)	22.85 (12.35)	21.75 (10.30)	0.100
Cholesterol (mg/d)	249.11 (155.38)	241.98 (138.79)	0.225
Saturated fat (g/d)	16.82 (9.02)	16.15 (7.73)	0.090
MUFA (g/d)	16.89 (9.98)	16.72 (8.79)	0.619
PUFA (g/d)	15.62 (9.49)	15.22 (8.58)	0.444
Magnesium (mg/d)	265.91 (125.54)	256.28 (106.15)	**0.001**
Zinc (mg/d)	7.84 (3.63)	7.44 (3.11)	**0.001**
Calcium (mg/d)	779.19 (417.30)	738.12 (341.87)	**<0.001**
Selenium (mg/d)	0.09 (0.04)	0.08 (0.04)	0.071
**HBI components**			
HBI water (cc)	938.98 (483.37)	907.13 (426.02)	**0.001**
HBI coffee tea (cc)	230.32 (236.42)	230.00 (231.42)	**0.044**
HBI low-fat milk (cc)	110.58 (189.04)	107.51 (183.21)	0.741
HBI fruit juice (cc)	1.89 (11.34)	1.26 (7.56)	0.146
HBI high-fat milk (cc)	65.53 (182.23)	65.53 (82.23)	**0.011**
HBI SSB (cc)	33.86 (70.26)	24.10 (61.75)	**<0.001**
HBI total beverage energy (kcal)	115.39 (117.09)	106.88 (96.43)	**0.004**
HBI met. Fluid. requirements	1344.01 (791.19)	1258.06 (680.58)	**<0.001**

Using Mann–Whitney for abnormal continuous variables.

Values are median (IQR).

Compared with the first tertile, there was a significant association between the last tertile of HBI and a lower odds ratio (OR) for MetS (OR = 0.66, 95% CI: 0.54–0.81; *P* < 0.001). After adjustment for confounding factors, the third tertile of HBI was significantly associated with a 40% lower risk for MetS compared with baseline (OR = 0.60, 95% CI: 0.48–0.74, *P* < 0.001) (Table 3). In Table 4, participants in the second tertile of HBS had a 30% lower risk for MetS compared with the first tertile. After adjustment for confounding factors, higher HBS in the second and third tertiles was significantly associated with lower odds of MetS compared with baseline (second tertile: OR = 0.64, 95% CI: 0.52–0.79, *P* < 0.001; third tertile: OR = 0.80, 95% CI: 0.65–0.97, *P* = 0.030).

**Table 3.** Associations between study variables with healthy beverage index in total population

	**Metabolic syndrome** Total population (*n* = 3380)			
Variables	Univariate OR (95%CI)	*P*-value	Multivariate OR (95%CI)	*P*-value
**HBI**				
HBI T1	Ref	–	Ref	–
HBI T2	0.88 (0.73–1.05)	0.175	0.83 (0.68–1.00)	0.059
HBI T3	**0.66 (0.54–0.81)**	**>0.001**	**0.60 (0.48–0.74)**	**>0.001**
Age (year)	1.03 (1.02–1.04)	**>0.001**	1.03 (1.02–1.04)	**>0.001**
BMI (kg/m^2^)	1.18 (1.15–1.20)	**>0.001**	1.18 (1.16–1.21)	**>0.001**
Physical activity (Met/week)	1.00 (1.00–1.00)	0.889	–	–
Energy (Kcal)	1.00 (1.00–1.00)	**>0.001**	–	–
**Gender**				
Male	Ref	–	Ref	–
Female	0.51 (0.43–0.59)	**>0.001**	0.51 (0.43–0.61)	**>0.001**
**SES**				
Low	Ref	–	Ref	–
Moderate	0.84 (0.70–1.01)	0.074	–	–
High	0.79 (0.65–0.95)	**0.015**	–	–
**Smoke exposure**				
No	Ref	–	Ref	–
Yes	1.66 (1.30–2.12)	**>0.001**	–	–
**Opium use**				
No	Ref	–	Ref	–
Yes	1.21 (0.64–2.28)	0.545	–	–
**Alcohol use**				
No	Ref	–	Ref	–
Yes	1.60 (1.12–2.27)	**0.009**	1.51 (1.04–2.20)	**0.030**
**Education**				
Diploma or less	Ref	–	Ref	–
Upper diploma	0.72 (0.55–0.94)	**0.017**	–	–
**Medication or Supplementation**				
No	Ref	–	Ref	–
Yes	0.76 (0.65–0.89)	**0.001**	–	–

SES, socioeconomic status.

Missing values in each variable were excluded from the analyses.

Using backward LR method for multivariate analysis.

Significant values are shown in bold.

Confounders: Age, BMI, Energy, Gender, SES, Smoke exposure, Alcohol use, Education, Medication or Supplementation.

**Table 4.** Associations between study variables with healthy beverage Score in total population

	Metabolic syndrome Total population (*n* = 3380)			
Variables	Univariate OR (95% CI)	*P*-value	Multivariate OR (95% CI)	*P*-value
**HBS**				
HBS T1	Ref	–	Ref	–
HBS T2	**0.70 (0.58–0.85)**	>**0.011**	**0.64 (0.52–0.79)**	**>0.001**
HBS T3	0.88 (0.73–1.06)	0.197	**0.80 (0.65–0.97)**	**0.030**
Age (year)	1.03 (1.02–1.04)	**>0.001**	1.034 (1.022–1.046)	**>0.001**
BMI	1.18 (1.15–1.20)	**>0.001**	1.185 (1.161–1.210)	**>0.001**
Physical activity (Met/week)	1.00 (1.00–1.00)	0.889	–	–
Energy (Kcal)	1.00 (1.00–1.00)	**>0.001**	–	–
**Gender**				
Male	Ref	–	Ref	–
Female	0.51 (0.43–0.59)	**>0.001**	0.50 (0.42–0.59)	**>0.001**
**SES**				
Low	Ref	–	Ref	–
Moderate	0.84 (0.70–1.01)	0.074	–	–
High	0.79 (0.65–0.95)	**0.015**	–	–
**Smoke exposure**				
No	Ref	–	Ref	–
Yes	1.66 (1.30–2.12)	**>0.001**	–	–
**Opium use**				
No	Ref	–	Ref	–
Yes	1.21 (0.64–2.28)	0.545	–	–
**Alcohol use**				
No	Ref	–	Ref	–
Yes	1.60 (1.12–2.27)	**0.009**	1.50 (1.03–2.19)	**0.033**
**Education**				
Diploma or less	Ref	–	Ref	–
Upper diploma	0.72 (0.55–0.94)	0.017	–	–
**Medication or Supplementation**				
No	Ref	–	Ref	–
Yes	0.76 (0.65–0.89)	**0.001**	–-	–

SES, socioeconomic status.

Missing values in each variable were excluded from the analyses.

Using backward LR method for multivariate analysis.

Adjusted for variables with *P* value <0.25 in univariate analysis.

Significant values are shown in bold.

Confounders: Age, BMI, Energy, Gender, SES, Smoke exposure, Alcohol use, Education, Medication or Supplementation.

The association between HBI tertiles and MetS components was assessed. After adjustment for confounding factors, the association between the third tertile of HBI and lower levels of Blood pressure and WC remained significant (OR = 0.64, 95% CI: 0.52–0.79, *P* < 0.001) (OR = 0.70, 95% CI: 0.54–0.91, *P* = 0.009) (Supplemental Table 1). Supplemental Table 2 shows data on each component of the MetS and their relationship with HBS. Overall, there was a significant association between the second tertile of HBS and a 21% lower risk of hypertension compared with baseline (OR = 0.79, 95% CI: 0.64–0.97, *P* = 0.025).

## Discussion

This study examined 3,380 employees of SUMS and found that higher adherence to healthy beverage patterns, as measured by HBI and HBS, was associated with a lower risk of MetS. In addition, higher HBI and HBS levels both were associated with lower blood pressure levels, and only higher HBI was associated with lower WC.

In line with findings of the current study, a previous research have shown that consuming healthy beverages such as water, tea, coffee, and low-fat milk and reducing the consumption of SSB can reduce the risk of MetS.^(29,30)^ However, in this study, the overall beverage pattern was assessed using the HBI and HBS which represent the quality and quantity of beverage intake based on nutrient content. A recent systematic review and meta-analysis by A.Cabrejas et al. found a positive association between higher consumption of SSB and increased risk of MetS.^(31)^ Also, a study by J.Mattei et al. demonstrated that replacing one serving of soda with homemade fruit juice could reduce the likelihood of MetS by 30% in the Hispanic population.^(32)^ In addition, a cohort study with a 6-year follow-up found that moderate coffee consumption may be associated with a lower risk of MetS.^(33)^ However, another cohort study with 14 years of follow-up showed no significant association between coffee, tea, alcohol, and diet cola consumption and MetS risk.^(34)^ Another study discovered that elevated HBI were linked to higher risk of MetS among men, whereas a reduced risk of MetS was observed among women; and the researchers proposed that the identical beverages may fulfil distinct functions in males and females.^(35)^ As you can see, there is still no consensus on the specific effects of different beverages on the MetS, and it is important to consider the complex interactions between different beverage types and their effects on health. The beneficial effects of healthy beverage patterns on the MetS can be attributed to several mechanisms. First, lower consumption of SSB has been consistently associated with decreased risk of obesity, CVD, and MetS in previous studies.^(36)^ Second, higher consumption of low-fat milk has been associated with a preventive effect against MetS.^(29)^ Third, lower total energy intake from beverages may reduce the risk of MetS and its components.^(37)^

Also, the present study has unveiled a noteworthy correlation between higher adherence to HBI and HBS with diminished blood pressure levels. The implications of these findings are particularly significant, given that hypertension is a prominent risk factor for CVD, the primary cause of global mortality.^(38)^ This observation aligns with previous research that has underscored the salutary impact of specific beverages on blood pressure regulation.^(25)^ For instance, a comprehensive meta-analysis of randomised controlled trials has revealed that the consumption of green tea significantly mitigates both systolic and diastolic blood pressure.^(39)^ In a prospective study conducted by L.Chen et al., it was discerned that a reduction in the intake of SSBs by one serving is correlated with a 1.8 mmHg decrease in systolic blood pressure and a 1.1. mmHg decrease in diastolic blood pressure in adults with prehypertension or stage 1 hypertension^(40)^ On the other hand, a meta-analysis of cohort studies has indicated an absence of significant association between coffee consumption and hypertension risk.^(41)^ An intriguing outcome of a study by A.Miranda et al. posits that the beneficial effects of coffee consumption against hypertension are exclusive to the non-smoking demographic.^(42)^ The potential mechanisms underpinning the positive influence of higher adherence to HBI and HBS on blood pressure encompass the presence of bioactive compounds, such as polyphenols and caffeine. These compounds have been demonstrated to enhance endothelial function, attenuate inflammation, and stimulate vasodilation.^(16)^ Another plausible explanation pertains to hydration status, which is recognised as a crucial determinant in blood pressure regulation. Increased consumption of water and other low-sugar beverages may facilitate the release of atrial natriuretic peptide (ANP) and augment right atrium pressure.^(43)^ Furthermore, the intake of natural fruit juice, rich in magnesium and potassium, can contribute to blood pressure reduction.^(35)^ Beyond these specific beverages, the overall quality and quantity of an individual’s beverage consumption may also influence blood pressure regulation because, it is possible that the components of HBI or HBS exert synergistic effects with each other.

This study also found a negative association between higher adherence to HBI and WC, indicating healthier body composition and lower risk of chronic diseases such as diabetes and CVD.^(44)^ This finding is consistent with other studies that have reported a protective effect of higher adherence to HBI against abdominal obesity.^(45,46)^ Conversely, a national cross-sectional study in Chinese children and adolescents found no significant association between SSBs consumption and overall obesity.^(47)^ One possible explanation for the inverse association between HBI and WC is that healthy beverages are usually low in calories and sugar, which may help people maintain a healthy weight and prevent central obesity. In addition, consumption of healthy beverages such as water and tea may promote feelings of satiety^(48)^ and reduce consumption of high-calorie sugary beverages, which are known to contribute to weight gain and obesity.^(49)^

This study has several strengths, including novelty in choosing the title, access to a large and valid sample of a cohort study, adjustment for confounding factors, and assessment of the MetS and its individual components. However, there are limitations to note. First, the cross-sectional design prevents the identification of a cause-and-effect relationship. Second, the food intake frequency questionnaire relies on participants’ long-term memory, which may introduce recall bias. Finally, while our research design adhered to rigorous methodologies and employed standardised procedures for data collection and analysis, inherent biases or confounding factors may have influenced the outcomes. Additionally, the study population comprised individuals from a specific demographic or clinical setting, which may limit the generalisability of our results to the broader population.

## Conclusion

In conclusion, a higher proportion of healthy beverage patterns, as measured by HBI and HBS, was associated with lower MetS risk, particularly lower blood pressure levels. Further studies are needed to confirm the association of HBI and HBS with MetS in different populations.

## Supporting information

Leilami et al. supplementary material Leilami et al. supplementary material

## Supplementary material

The supplementary material for this article can be found at https://doi.org/10.1017/jns.2024.65
