# Supplementary material for: Association between healthy beverage index and healthy beverage score with metabolic syndrome: a cross-sectional study
Source: J Nutr Sci. 2025 Feb 24;14:e19. doi: 10.1017/jns.2024.65 (PMC11867817; doi:10.1017/jns.2024.65)
Supplement: Leilami et al. supplementary material [file S204867902400065Xsup001.docx]

**Supplementary Table 1.** Associations between study variables with healthy beverage index in total population in multivariate analysis.

| **Low HDL-C^5^** | | **High Triglyceride^4^** | | **High Waist Circumference^3^** | | **High fasting blood glucose^2^** | | **High Blood Pressure^1^** | | **Metabolic Syndrome Components** | |
| --- | --- | --- | --- | --- | --- | --- | --- | --- | --- | --- | --- |
| P-  Value | Multivariate  OR (95%CI) | P-  Value | Multivariate  OR (95%CI) | P-  Value | Multivariate  OR (95%CI) | P-  Value | Multivariate  OR (95%CI) | P-  Value | Multivariate  OR (95%CI) | Variables | |
| - | Ref | - | Ref | - | Ref | - | Ref | - | Ref | HBI T1 | |
| **0.017** | **1.24**  **(1.40-1.48)** | - | - | 0.084 | 1.23  (0.97-1.57) | - | - | 0.282 | 0.90  (0.74-1.09) | HBI T2 | |
| 0.285 | 0.90  (0.74-1.08) | - | - | **0.009** | **0.70**  **(0.54-0.91)** | - | - | **0.001>** | **0.64**  **(0.52-0.79)** | HBI T3 | |
| 0.063 | 1.01  (0.99-1.02) | - | - | **0.008** | **1.02**  **(1.00-1.03)** | - | - | **0.001>** | **1.05**  **(1.03-1.06)** | Age (year) | |
| - | - | - | - | **<0.001** | **2.01**  **(1.91-2.12)** | - | - | **0.001>** | **1.14**  **(1.12-1.16)** | BMI (Kg/m^2^) | |
| - | - | - | - | - | - | **0.024** | **1.00**  **(1.00-1.00)** | - | - | Physical activity (Met/week) | |
| - | - | - | - | 0.052 | 1.00  (1.00-1.00) | - | - | - | - | Energy (Kcal) | |
| - | Ref | - | Ref | - | Ref | - | Ref | - | Ref | Male | Gender |
| **0.001>** | **0.18**  **(0.15-0.21)** | - | - | **0.001>** | **31.47**  **(23.57-42.02)** | - | - | **0.001>** | **0.18**  **(0.15-0.22)** | Female |  |
| - | Ref | - | Ref | - | Ref | - | Ref | - | Ref | low | SES |
| - | - | - | - | 0.094 | 0.80  (0.63-1.03) | 0.198 | 0.87  (0.70-1.07) | - | - | Moderate |  |
| - | - | - | - | 0.422 | 1.11  (0.86-1.43) | **<0.001** | **0.65**  **(0.51-0.81)** | - | - | High |  |
| - | Ref | - | Ref | - | Ref | - | Ref | - | Ref | No | Smoke exposure |
| - | - | - | - | - | - | 0.056 | 1.32  (0.99-1.76) | - | - | Yes |  |
| - | Ref | - | Ref | - | Ref | - | Ref | - | Ref | No | Opium use |
| - | - | **0.030** | **0.44**  **(0.21-0.92)** | - | - | - | - | - | - | Yes |  |
| - | Ref | - | Ref | - | Ref | - | Ref | - | Ref | No | Alcohol use |
| - | - | 0.116 | 1.46  (0.93-1.85) | **0.006** | **1.98**  **(1.21-3.25)** | - | - | - | - | Yes |  |
| - | Ref | - | Ref | - | Ref | - | Ref | - | Ref | Diploma  or less | Education |
| - | - | **0.036** | **1.11**  **(1.02-2.08)** | - | - | - | - | - | - | Higher  diploma |  |
| - | Ref | - | Ref | - | Ref | - | Ref | - | Ref | No | Medication  Supplementation |
| - | - | - | - | - | - | - | - | **0.016** | **0.79**  **(0.66-0.95)** | Yes |  |

SES, socioeconomic status.

Missing values in each variable were excluded from the analyses.

Using Backward LR method for Multivariate analysis.

Significant values are shown in bold.

1. Adjusted for HBI, age, BMI, PA, Energy, gender, SES, Smoke exposer, opium use, alcohol use, education, medication supplementation.
2. Adjuster for age, PA, SES.
3. Adjusted for HBI, age, BMI, PA, energy, gender, SES, smoke exposer, alcohol use, education, medication supplementation.
4. Adjusted for gender, opium use, medication supplementation.
5. Adjusted for HBI, age, PA, Energy, gender, Smoke exposer, opium use, alcohol use, education, medication supplementation.

**Supplementary Table 2.** Associations between study variables with healthy beverage score in total population in multivariate analysis.

| **Low HDL-C^5^** | | **High Triglyceride^4^** | | **High Waist Circumference^3^** | | **High fasting blood glucose^2^** | | **High Blood Pressure^1^** | | **Metabolic Syndrome Components** | |
| --- | --- | --- | --- | --- | --- | --- | --- | --- | --- | --- | --- |
| P-  Value | Multivariate  OR (95%CI) | P-  Value | Multivariate  OR (95%CI) | P-  Value | Multivariate  OR (95%CI) | P-  Value | Multivariate  OR (95%CI) | P-  Value | Multivariate  OR (95%CI) | Variables | |
| - | Ref | - | Ref | - | Ref | - | Ref | - | Ref | HBS T1 | |
| - | - | - | - | - | - | - | - | **0.025** | **0.79**  **(0.64-0.97)** | HBS T2 | |
| - | - | - | - | - | - | - | - | 0.239 | 0.88  (0.72-1.08) | HBS T3 | |
| 0.062 | 1.01  (0.99-1.02) | - | - | **0.010** | **1.02**  **(1.00-1.03)** | - | - | **0.001>** | **1.05**  **(1.03-1.06)** | Age (year) | |
| - | - | - | - | **<0.001** | **2.00**  **(1.90-2.10)** | - | - | **0.001>** | **1.14**  **(1.11-1.16)** | BMI (Kg/m^2^) | |
| - | - | - | - | - | - | **0.024** | **1.00**  **(1.00-1.00)** | - | - | Physical activity (Met/week) | |
| - | - | - | - | **0.015** | **1.00**  **(1.00-1.00)** | - | - | - | - | Energy (Kcal) | |
| - | Ref | - | Ref | - | Ref | - | Ref | - | Ref | Male | Gender |
| **0.001>** | **0.18**  **(0.15-0.21)** | - | - | **0.001>** | **30.49**  **(22.89-42.60)** | - | - | **0.001>** | **0.18**  **(0.14-0.21)** | Female |  |
| - | Ref | - | Ref | - | Ref | - | Ref | - | Ref | low | SES |
| - | - | - | - | 0.090 | 0.80  (0.63-1.03) | 0.198 | 0.87  (0.70-1.07) | - | - | Moderate |  |
| - | - | - | - | 0.420 | 1.11  (0.86-1.43) | **<0.001** | **0.65**  **(0.51-0.81)** | - | - | High |  |
| - | Ref | - | Ref | - | Ref | - | Ref | - | Ref | No |  |
| - | - | - | - | - | - | 0.056 | 1.32  (0.99-1.76) | - | - | Yes | Smoke  Exposure |
| - | Ref | - | Ref | - | Ref | - | Ref | - | Ref | No | Opium use |
| - | - | **0.036** | **0.45**  **(0.22-0.91)** | - | - | - | - | - | - | Yes |  |
| - | Ref | - | Ref | - | Ref | - | Ref | - | Ref | No | Alcohol use |
| - | - | **0.036** | **1.46**  **(1.02-2.08)** | **0.009** | **1.91**  **(1.17-3.11)** | - | - | - | - | Yes |  |
| - | Ref | - | Ref | - | Ref | - | Ref | - | Ref | Diploma  or less | Education |
| - | - | - | - | - | - | - | - | - | - | Higher  diploma |  |
| - | Ref | - | Ref | - | Ref | - | Ref | - | Ref | No | Medication  Supplementation |
| - | - | - | - | - | - | - | - | **0.019** | **0.80**  **(0.66-0.96)** | Yes |  |

SES, socioeconomic status.

Missing values in each variable were excluded from the analyses.

Using Backward LR method for Multivariate analysis.

Significant values are shown in bold.

1. Adjusted for HBS, age, BMI, PA, Energy, gender, SES, Smoke exposer, opium use, alcohol use, education, medication supplementation.
2. Adjusted for HBS, age, PA, SES, Smoke exposer.
3. Adjusted for HBS, age, BMI, PA, Energy, gender, SES, Smoke exposer, opium use, alcohol use, education, medication supplementation.
4. Adjusted for HBS, gender, opium use, alcohol use, medication supplementation.
5. Adjusted for HBS, age, PA, Energy, gender, SES, Smoke exposer, opium use, alcohol use, education, medication supplementation.
